# The G2-to-M transition from a phosphatase perspective: a new vision of the meiotic division

**DOI:** 10.1186/s13008-020-00065-2

**Published:** 2020-05-25

**Authors:** Tom Lemonnier, Aude Dupré, Catherine Jessus

**Affiliations:** Laboratoire de Biologie du Développement-Institut de Biologie Paris Seine, LBD-IBPS, Sorbonne Université, CNRS, 75005 Paris, France

**Keywords:** Cell division, Meiotic division, Oocyte, Protein phosphatases, Protein kinases, Protein phosphorylation, PP2A, Cdk1, PKA

## Abstract

Cell division is orchestrated by the phosphorylation and dephosphorylation of thousands of proteins. These post-translational modifications underlie the molecular cascades converging to the activation of the universal mitotic kinase, Cdk1, and entry into cell division. They also govern the structural events that sustain the mechanics of cell division. While the role of protein kinases in mitosis has been well documented by decades of investigations, little was known regarding the control of protein phosphatases until the recent years. However, the regulation of phosphatase activities is as essential as kinases in controlling the activation of Cdk1 to enter M-phase. The regulation and the function of phosphatases result from post-translational modifications but also from the combinatorial association between conserved catalytic subunits and regulatory subunits that drive their substrate specificity, their cellular localization and their activity. It now appears that sequential dephosphorylations orchestrated by a network of phosphatase activities trigger Cdk1 activation and then order the structural events necessary for the timely execution of cell division. This review discusses a series of recent works describing the important roles played by protein phosphatases for the proper regulation of meiotic division. Many breakthroughs in the field of cell cycle research came from studies on oocyte meiotic divisions. Indeed, the meiotic division shares most of the molecular regulators with mitosis. The natural arrests of oocytes in G2 and in M-phase, the giant size of these cells, the variety of model species allowing either biochemical or imaging as well as genetics approaches explain why the process of meiosis has served as an historical model to decipher signalling pathways involved in the G2-to-M transition. The review especially highlights how the phosphatase PP2A-B55δ critically orchestrates the timing of meiosis resumption in amphibian oocytes. By opposing the kinase PKA, PP2A-B55δ controls the release of the G2 arrest through the dephosphorylation of their substrate, Arpp19. Few hours later, the inhibition of PP2A-B55δ by Arpp19 releases its opposing kinase, Cdk1, and triggers M-phase. In coordination with a variety of phosphatases and kinases, the PP2A-B55δ/Arpp19 duo therefore emerges as the key effector of the G2-to-M transition.

## Background

Cells proliferate by means of the mitotic cell cycle, a process underlying growth, development and renewal in all eukaryotic organisms. Understanding the control of cell division is therefore pivotal to diverse and essential biological problems and has thus fascinated biologists since the middle of the nineteenth century. Decades of studies were dedicated to the description of the dramatic cell restructuration orchestrating the mechanics of M-phase, i.e., the proper segregation of chromosomes into two daughter cells, relying on chromosome condensation within nuclei, their splitting to produce a pair of sister chromatids, and movement of each sister chromatid to opposite poles of the cell thanks to the dynamics of the microtubular mitotic spindle and the control of sister chromatid cohesion. While the mechanics of mitosis makes the cell cleavage possible, specific regulatory mechanisms must tell the cell when to embark on M-phase. For this reason, one of the holy grails in this field has been the identification of factors that trigger the onset of mitosis. The symbiosis between yeast genetics and frog biochemistry led to an extraordinary progress in this issue in the 80s [1]. These pioneered complementary approaches revealed that entry into mitosis depends on the activation of the M-phase Promoting Factor (or MPF), the universal mitotic inducer in eukaryotic cells, consisting of the Cdk1-Cyclin B complex and the Greatwall kinase [2].

**Fig. 1:**
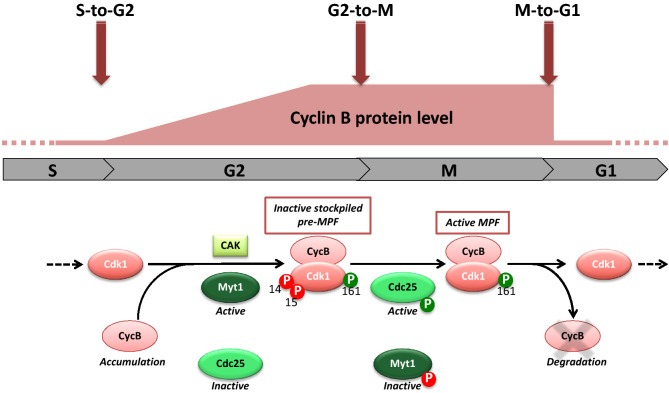
The three transitions of the mitotic cycle involving Cdk1-Cyclin B. During the G2-phase, Cyclin B accumulates and binds to Cdk1 that is phosphorylated at T161 by CAK and at Y15 and T14 by Wee1/Myt1. The complex is thus inactive. At the G2-to-M transition, Wee1/Myt1 are turned off while Cdc25 is turned on, promoting Y15 and T14 dephosphorylations and Cdk1 activation. During M-phase, Cdk1-Cyclin B phosphorylates many mitotic substrates, including Wee1/Myt1 and Cdc25. At the end of M-phase, MPF activates the Cyclin proteolytic degradation system, thus inducing its own inactivation

This great discovery led to a new orthodoxy whereby entry into mitosis occurs when a set of substrate proteins implicated in mitosis progression changes their state of phosphorylation that is brought about by the activity of the Cdk1-Cyclin B kinase. The regulation of this mitosis master kinase was based on the following global scheme (Fig. 1) [3]. At the end of the DNA replication phase (S-phase), the cell enters a G2-phase during which Cyclin B accumulates and binds to Cdk1 that is expressed at a constant level throughout the cell cycle. Cdk1 is phosphorylated at T161, triggering a refolding of the active site cleft that allows the enzyme to bind substrates. At the same time, a double phosphorylation of Cdk1, at Y15 and T14, achieved by two partly redundant kinases, Wee1 and Myt1, maintains the Cdk1-Cyclin B complex under an inactive state, known as pre-MPF.

At the end of the G2-phase, new Cyclin B synthesis is not required anymore, all components needed for mitosis are present but MPF is inactive. Maintaining MPF inactive all over the G2-phase generates a time window during which the cell prepares itself for mitosis and performs quality control of damaged or unreplicated DNA, repairing errors before further progression through the cell cycle. The G2-to-M transition is sudden and irreversible, relying on the sharp activation of Cdk1-Cyclin B complexes accumulated during the G2-phase: the inhibitory Y15 and T14 phosphorylations are removed, because the Y15-T14 kinases, Wee1/Myt1, are turned off while the Y15-T14 phosphatase, Cdc25, is turned on (Fig. 1) [4]. Exactly how this system is normally regulated such that cells do not enter mitosis while DNA replication is in progress or DNA is damaged is not so clear. Phosphorylation of Cdc25 by Chk1 (Checkpoint kinase 1), downstream of ATM (Ataxia-Telengiectasia mutated), seems to be crucial [5]. However, what keeps Wee1 and Myt1 active and inhibits Cdc25 in order to stop Cdk1 from turning on prematurely during undamaged S- and G2-phases is not well understood. Nor is it clear what changes at the end of the G2-phase, to flip the switch to allow the entry into mitosis. Cyclin A would be instrumental as a triggering factor, promoting Wee1 phosphorylation and priming mitotic entry through Cyclin B [6–10]. Activated Cdk1-Cyclin B phosphorylates many mitotic substrates, including Wee1/Myt1 and Cdc25. Phosphorylating these Y15-T14-modifying enzymes makes the system auto-catalytic, resulting in a fast and bistable switch indicative of the rapidity and irreversibility that characterize entry into mitosis. Besides its own regulators, Cdk1 phosphorylates hundreds of proteins involved in the structural changes during mitosis, as well as regulatory kinases that in turn lead to phosphorylations of other mitotic substrates. This huge wave of protein phosphorylations brings about processes such as nuclear envelope breakdown, chromosome condensation and spindle assembly. After the cytoskeleton has been reorganized for division and the condensed chromosomes aligned on the metaphase plate, MPF activates the Cyclin proteolytic degradation system, thus inducing anaphase and its own inactivation (Fig. 1). The remaining steps of cell division, such as cytokinesis, occur as mitotic phosphorylations are reversed after MPF inactivation.

At the end of the twentieth century, the cell cycle was viewed as regulated only by two functionally different levels of the master kinase, MPF, either high or low, the change between them being very rapid. An offensive view proposed that these two levels of MPF would define two cell cycle states: cells with high levels of MPF are in mitosis, and those with low levels of MPF are in interphase [3, 11]. Controlling mitosis entry was envisaged as a matter of protein kinases, with the idea that phosphatase activities were rather constant and not tightly regulated, overwhelmed by kinase up-regulations and revealed by the inactivation of the same kinases. A new vision progressively emerged about 10 years ago, supported by genetic, biochemical and reverse genetic approaches: as Cdk activity is regulated by multiple mechanisms to promote cell cycle progression, one or more antagonizing phosphatase(s) are probably regulated in the opposite sense. This was undoubtedly confirmed with the discovery that a specific phosphatase, PP2A-B55 (B55δ in *Xenopus* [12], B55α or B55δ in mice [13] and B55α in human [14]), carries the major activity against Cdk1 regulators and its mitotic substrates. Its activity fluctuates during the cell cycle, being high in interphase and low in mitosis, controlling Cdk1 activation and M-phase entry as well as mitotic progression [15]. Hence, the cell cycle, and especially the control of mitosis, was no longer viewed as under the unique control of the master kinase, Cdk1: protein phosphatases are obviously as important as kinases. However, our knowledge about phosphatases in controlling M-phase entry lags well behind that of kinases. This chapter is focused on our current understanding of the phosphatases that are important in the control of Cdk1 activation and M-phase entry, based on a specific powerful model system, the resumption of oocyte meiotic division, that has served as an historical model to decipher the molecular controls of the G2-to-M transition of the cell cycle.

**Fig. 2:**
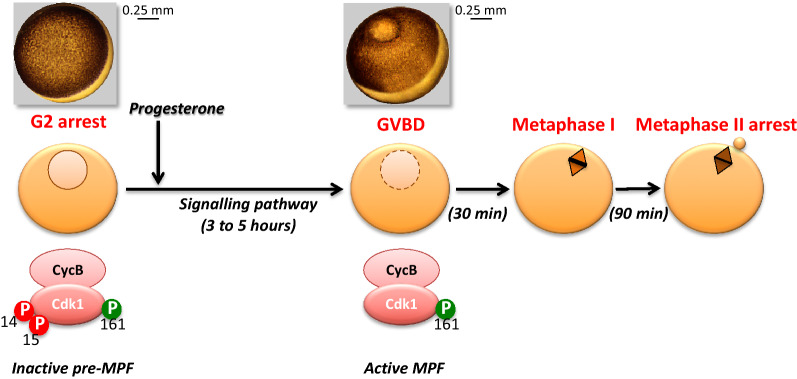
Resumption of *Xenopus* oocyte meiosis. G2-arrested oocytes contain pre-MPF, i.e. inactive Cdk1-Cyclin B complexes in which Cdk1 is inhibited by T14 and Y15 phosphorylation. Progesterone initiates a signalling pathway that leads to T14-Y15 dephosphorylation of Cdk1. MPF promotes the breakdown of the nuclear envelope (GVBD for germinal vesicle breakdown, identified by a white spot at the pole of the brown hemisphere) and formation of the metaphase I spindle. After extrusion of the first polar body, the metaphase II spindle is organized and the oocyte arrests at this stage, until fertilization. Top: two pictures of *Xenopus* oocytes, G2 arrest (left) and GVBD (right)

Oocytes are indeed simple and powerful experimental systems characterized by natural cell cycle arrest points released by defined stimuli to induce cell cycle progression. During oogenesis, oocytes enter meiosis and stop in prophase of the 1st meiotic division. This universal arrest lasts for an extraordinary long period during the lifetime of the female (more than 40 years in humans), and covers the period of oocyte growth in preparation for embryonic development. In amphibians as *Xenopus*, at the end of this 2-to-3-year-long period, the oocytes are huge yolky cells with a large nucleus, called germinal vesicle (Fig. 2). The prophase arrest of these full-grown oocytes resembles a late G2-phase. Indeed, although these cells have already entered into M-phase by performing the critical steps of prophase I and condensing chromosomes, their cell cycle arrests with a high level of cytoplasmic inactive Cdk1-Cyclin B complexes, pre-MPF, in which Cdk1 has been phosphorylated at T14-Y15 inhibitory sites, reminiscent of the end of somatic G2-phase. Release of this G2-like arrest at the time of ovulation is induced by external stimuli, the steroid hormone progesterone in *Xenopus*, which initiates a signal transduction pathway whose ultimate goal is the activation of Cdk1-Cyclin B complexes (Fig. 2).

Once activated, MPF promotes entry into the first meiotic division: breakdown of the nuclear envelope (known as GVBD for germinal vesicle breakdown), formation of the metaphase I spindle and emission of the first polar body at the exit of meiosis I. Then, the oocyte immediately proceeds to the 2nd meiotic division, without intervening S-phase between meiosis I and II, and arrests at the metaphase II stage due to the sustained stability of high MPF activity (Fig. 2).

Hence, meiosis resumption recapitulates the biochemical hallmarks of the G2-to-M transition control by MPF. *Xenopus laevis* oocytes are exceptionally suitable for biochemical experimental approaches to decipher the molecular control of this transition: oocytes are naturally arrested in a G2-like state in the ovary, from which these cells are easily recovered by manual dissection and maintained for 3 to 5 days under non-sterile conditions in a saline medium; adding progesterone in the external medium induces MPF activation that is assayed by scoring the appearance of a white spot in the middle of the brown pigmented hemisphere due to GVBD (Fig. 2); they are giant cells (1.2 mm in diameter) that are easily microinjected with various reagents (proteins, mRNA, anti-sense oligonucleotides, morpholinos, etc.); their huge amounts (thousands of them per ovary) as well as their high protein content (30 µg of soluble proteins per oocyte) allows biochemical and proteomic approaches. Thanks to the biochemistry of oocytes, and especially amphibian oocytes, a number of mitotic regulators have been discovered, starting with MPF itself [16–18].

**Fig. 3:**
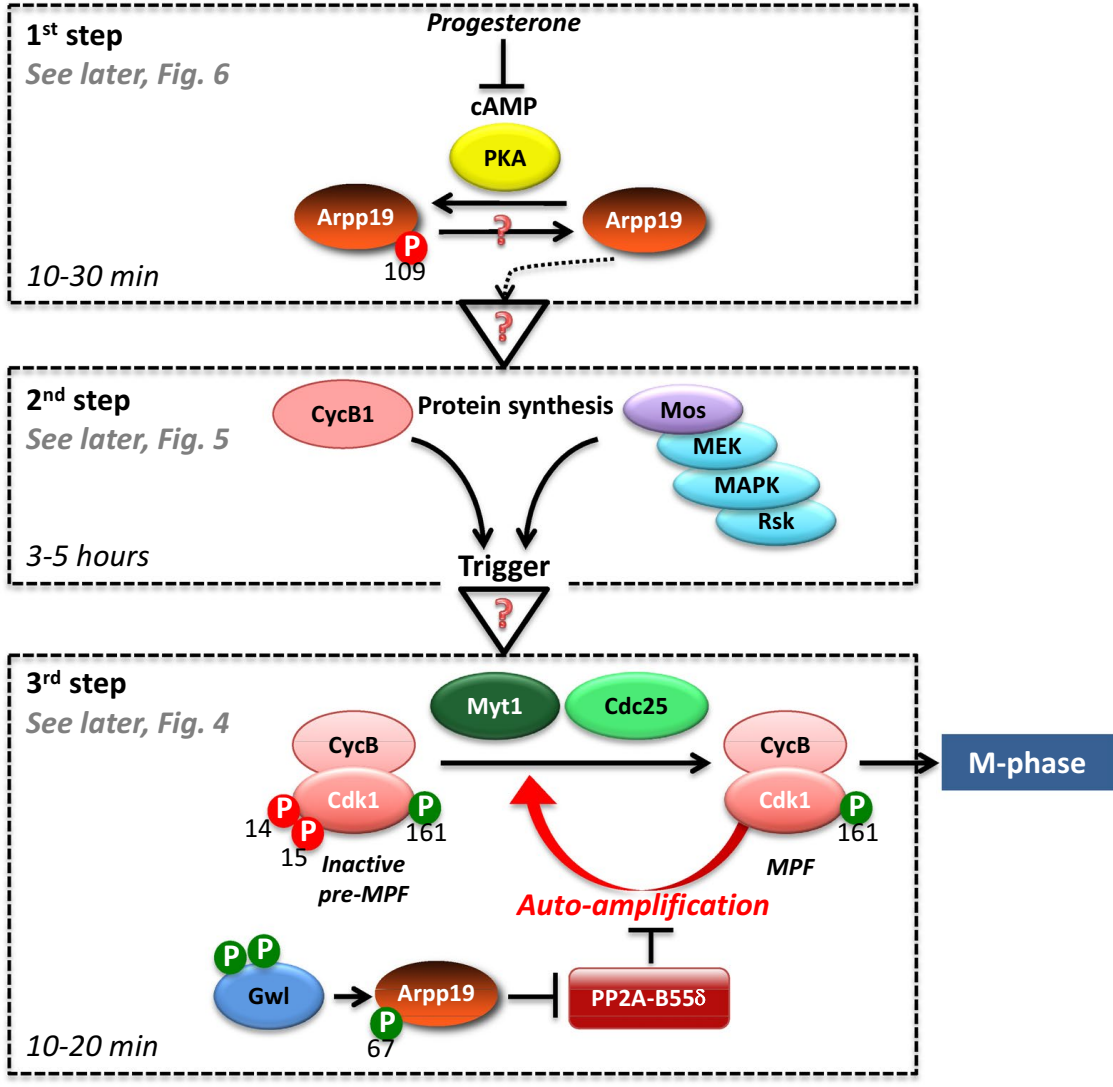
From progesterone to Cdk1 activation: the scattered elements of a not fully understood signalling pathway

In this model system, the G2-to-M transition can be considered as a 3-step process. G2-arrested oocytes contain a stockpile of inactive Cdk1-Cyclin B complexes, with Cdk1 being phosphorylated at T14 and Y15 by the Myt1 kinase (Wee1 is not expressed at the protein level in these cells [19]). Myt1 kinase is active and Cdc25 is inactive, due to their hypo-phosphorylated states. The first step corresponds to the reception and the integration of the external signal. Progesterone is the physiological hormone secreted by follicular cells that releases the G2 arrest [20]. It binds to membrane receptors whose identification has been an ongoing challenge for more than half a century. They probably correspond to both the conventional transcriptional progesterone receptor, which is partly located in membrane fractions [21, 22], and a membrane progestin receptor, unrelated to nuclear steroid receptors, but instead having some of the characteristics of G protein-coupled receptors [23]. Upon binding to progesterone, they inhibit adenylate cyclase activity. As a consequence, cAMP concentration drops within 10 to 30 min, leading to the inhibition of the cAMP-dependent protein kinase (PKA), and the consecutive dephosphorylation of critical PKA substrates, a necessary step for the G2 release (Fig. 3) [24–27]. In *Xenopus* oocyte, the small protein Arpp19 is the only well-characterized PKA substrate so far, whose dephosphorylation is required for the G2 release [28]. The second step is the molecular network triggered by PKA downregulation and Arpp19 dephosphorylation that leads to Cdk1 activation. The nature of the biochemical links that connect the inhibition of PKA and Arpp19 dephosphorylation, both occurring within 30 min after progesterone addition, with MPF activation 3 to 5 h later is a long-standing mystery. It includes however the synthesis of new proteins from mRNAs present in the oocyte cytoplasm, notably the translation of three proteins, Cyclin B, Ringo/Speedy and the kinase Mos that activates MEK, which in turn activates mitogen-activated protein kinase (MAPK) (Fig. 3) [29]. These newly synthesized proteins are required for the third step, the rapid activation of the stockpile of inactive Cdk1-Cyclin B complexes.

This last period that promotes an irreversible entry into the first meiotic division, relies on a 2-step mechanism: the formation of a “starter” amount of active MPF and then a positive feedback auto-amplification mechanism (Fig. 3). The coordinated action of the newly synthesized proteins initiates Myt1 inhibition and Cdc25 activation, each of them depending on their phosphorylation at multiple sites mainly achieved by Cdk1 [30, 31]. As a result, the conversion of the inactive Y15-T14-phosphorylated Cdk1-Cyclin B complexes into active MPF molecules starts. An autocatalytic system is then launched: more MPF is formed, more it promotes Cdc25 activation and Myt1 inhibition and more rapidly active MPF accumulates (Fig. 3). This key circuit controls the entry into both mitosis and meiosis.

This brief overview provides a picture of the G2-to-M transition, which is mainly orchestrated by kinases. A set of important results has recently changed this vision. Indeed, the Myt1–Cdc25–Cdk1 activation loop is inhibited by a specific S/T phosphatase, PP2A-B55δ, which likely leads either directly or indirectly to phosphates removal from phosphorylated Cdc25 and Myt1 [12, 32–35]. Inhibition of PP2A-B55δ is therefore necessary for Cdk1 activation. Its down-regulation is achieved by the protein Arpp19 that is phosphorylated by the protein kinase Greatwall (Gwl). This converts Arpp19 into a potent and specific inhibitor of PP2A-B55δ and allows the Cdk1 activating circuit to take place (Fig. 3) [34, 36, 37].

As we will see below, protein phosphatases have important roles all over meiosis resumption, which depends on a good coordination of phosphatases with opposing kinases. This is achieved through the control of phosphatases, a topic that has received little attention for a long time. Our aim is to highlight the critical roles played by various phosphatases and their specific regulation during the different steps of the oocyte G2-to-M transition. We propose to turn the clock back, starting with the positive feedback loop that allows the rapid conversion of pre-MPF to MPF, then addressing the generation of the MPF trigger that initiates this loop, and ending with the initial event: the PKA substrates whose dephosphorylation lays at the top of the signalling pathway.

### A powerful positive feedback in Cdk1 activation makes the M-phase commitment abrupt and irreversible

**Fig. 4:**
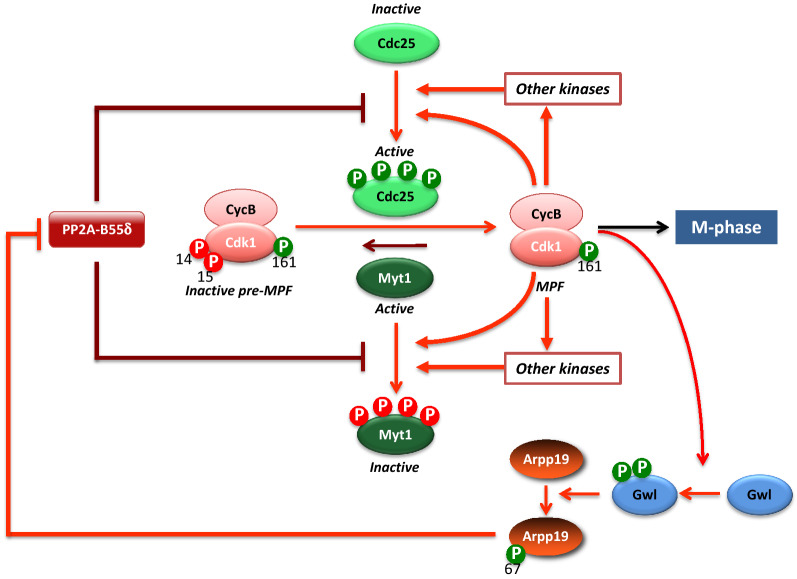
Molecular circuitry of the Cdk1 positive feedback loop. In G2-arrested oocytes, Cdk1-Cyclin B is stockpiled in an inactive state, with Cdk1 phosphorylated at T14 and Y15 by Myt1, while Cdc25 is inactive. PP2A-B55δ maintains Cdc25 and Myt1 under a dephosphorylated state. The first active molecules of Cdk1 reverse the balance by phosphorylating Myt1 and Cdc25. They also recruit other kinases that facilitate further phosphorylation of Myt1 and Cdc25. Finally, Cdk1 indirectly inactivates PP2A-B55δ through Gwl and S67-phosphorylated Arpp19

One major issue of the G2-to-M transition is to understand the details of the sharp activation of MPF, which involves series of feed-forward loops. At the center of this feedback mechanism, inhibition of protein phosphatases is critical. In G2-arrested oocytes, Cdk1-Cyclin B complexes are stockpiled in an inactive state, pre-MPF, with Cdk1 being inhibited by phosphorylation at T14 and Y15 under the control of the active kinase Myt1. Its counteracting enzyme, the phosphatase Cdc25, is inactive (see Sidebar A for the biochemical characteristics of Cdc25 phosphatases). Additionally, the activity of PP2A, likely the specific PP2A-B55δ isoform, maintains actively Cdc25 and Myt1 under a hypo-phosphorylated state, required for the inactivation of Cdc25 and the activity of Myt1 [12, 32–35, 38]. The feed-forward loop that activates Cdk1 relies on two capacities of Cdk1: first, to phosphorylate its own direct regulators, Myt1 and Cdc25; second, to indirectly inactivate its antagonizing protein phosphatase, PP2A-B55δ (Fig. 4). Hence, once this circuit is launched, the cell switches completely into M-phase in an abrupt and irreversible manner.

**Table:** 

**Sidebar A.** Cdc25 at a glance
Cdc25 is a dual specific phosphatase, which targets phosphorylated serine, threonine and tyrosine. It is a monomeric enzyme expressed in all eukaryotes, comprising several isoforms depending on species (from one in fungi to four in *Caenorhabditis elegans* and *Xenopus*, three in humans). Cdc25 proteins can be divided in two regions. The N-terminal regions are highly divergent in sequence, contain numerous sites for phosphorylation and ubiquitination, which are involved in regulating phosphatase activity, protein levels, association with other proteins and intracellular localization thanks to nuclear import–export signals. The C-terminal regions are more homologous and contain a small catalytic domain with the signature motif HCX5R of the Tyrosine-phosphatases but with a surprising lack of any apparent substrate recognition site. The active site face of the Cdc25s is astonishingly flat and the active site pocket is extremely shallow, well-suited for allowing access to both pT- and pY-containing substrates. Cdc25 phosphatases exhibit high specificity for pTpY-Cdk-Cyclin substrates, acting in a stepwise mechanism, wherein the enzyme dissociates after dephosphorylation of pT and must re-associate for dephosphorylation of pY [39].

### Reversal of the balance of Myt1 and Cdc25

Upon entry in mitosis or meiosis, Myt1 and Cdc25 are both regulated by phosphorylation at multiple residues but in opposite ways. Myt1 is hypophosphorylated and active in G2 and is inactivated by phosphorylation during meiosis resumption [33, 40]. Conversely, Cdc25 is hypophosphorylated and inactive in G2 and gets hyperphosphorylated and activated in M-phase [41–43]. Whether for Myt1 or Cdc25, these phosphorylations are mostly catalyzed by Cdk1, which thus regulates antagonistically the activity of its own regulators [30, 40, 44–49].

As a result, Cdk1 amplifies and maintains its own activity at a stable and high level to promote GVBD and the metaphase I spindle formation. Likewise, the efficiency of the Cdk1 activation process is enhanced by other kinases that directly contribute to the phosphorylation of Myt1 and Cdc25, accelerating T14 and Y15 dephosphorylation of Cdk1: the Polo-like kinase Plx1 [38, 46, 50, 51], Aurora A [52, 53], several kinases of the Mos/MAPK/p90^Rsk^ cascade, as Mos [54], MEK [55] and p90^Rsk^ [31, 56] and possibly others (Fig. 4). Cdk1 synergizes with these various kinases by phosphorylating sites in Myt1 and Cdc25 that facilitate the recruitment of these other kinases and further phosphorylation of Myt1 and Cdc25 [31, 57]. As expected for members of the Cdk1 auto-amplification loop, the injection in *Xenopus* G2-arrested oocytes of Cdc25, Cyclin B and all the various players of the loop under their active form, directly activates Cdk1 by reversing the balance of Myt1 and Cdc25 activities [58–66].

A specific phosphorylation of Cdc25 is worth mentioning. In somatic cells, Cdc25 activation is restrained by DNA-responsive checkpoints that prevent mitosis in the presence of incompletely replicated or damaged DNA. Checkpoint inhibition of Cdc25 is mediated largely through phosphorylation of Cdc25 at S287 (*Xenopus* numbering, S216 in human Cdc25C), which is required for docking of the small protein 14-3-3 [67–70]. 14-3-3 binding does not alter Cdc25 activity, but sequesters Cdc25 in the cytoplasm, preventing access to its substrate, Cdk1-Cyclin B [67, 71]. Although activation of DNA checkpoints is critical to block Cdc25, other non-checkpoint kinases also can phosphorylate S287 and may contribute to Cdc25 suppression during normal G2-phase. This is the case in the G2-arrested oocyte, which is devoid of checkpoints in response to DNA damage and stalled DNA replication but where Cdc25 is phosphorylated at S287 by PKA [72]. As described above, Cdc25 activation is accompanied by its phosphorylation at multiple residues, occurring through the Cdk1-Cyclin B mediated feed-forward loop [30, 41–43, 47]. As these activating phosphorylations take place, S287 phosphorylation is lost as well as Cdc25 binding to 14-3-3 [67, 71, 72]. It has been shown that the phosphatase responsible of S287 dephosphorylation is the protein phosphatase 1 (PP1, see Sidebar B for the biochemical characteristics of the PP1 phosphatase) [73, 74].

**Table:** 

**Sidebar B.** PP1 at a glance
PP1 is a multimeric complex composed of one catalytic subunit (PP1c) associated to one or two regulatory proteins, called PIPs (PP1-interacting proteins). In vertebrates, PP1c is expressed as four distinct isoforms: PP1α, β, γ1 and γ2, the latter one being specific of testis. In eukaryotes, more than 200 PIPs have been described to date and these proteins regulate substrate specificity and cell localization of PP1c. Some of them act as direct regulators of PP1 activity while others, either specify its subcellular localization or behave as pseudo-substrates to inhibit PP1 activity [75–77]. PIPs interact with PP1c through well-characterized domains, the SLiMs (short linear motifs). In particular, 85% of PIPs share one main binding motif corresponding to the RVXF consensus sequence, in which X represents any amino acid except proline [78]. Besides its regulation by PIPs, PP1c activity is directly inhibited by phosphorylation at T320 within its catalytic domain [79–81]. This residue is dephosphorylated by PP1c itself and phosphorylated by Cdk1 and Cdk2 during the cell cycle.

At time of Cdk1 activation, removal of 14-3-3 from Cdc25 precedes S287 dephosphorylation, a necessary prerequisite for the action of PP1 since 14-3-3 binding inhibits S287 dephosphorylation [73]. Moreover, S285 phosphorylation of Cdc25 by Cdk1 greatly enhances recruitment of PP1 to Cdc25, thereby accelerating S287 dephosphorylation and M-phase entry [74]. All these observations clearly indicate that, as the activating phosphorylations of Cdc25, S287 dephosphorylation by PP1 is included into the Cdk1-catalyzed positive feedback loop.

Moreover, they point to a positive function played by PP1 for the running of the loop, a conclusion that is difficult to reconcile with various results showing that either PP1 is dispensable for the Cdk1 auto-activation circuit, or even plays a negative role in this system. In *Xenopus* oocyte extracts recapitulating the auto-amplification mechanism, Cdk1 is directly activated by PP2A depletion and is not affected by the down- or up-regulation of PP1 activity, leading to the conclusion that PP2A is the key phosphatase that negatively regulates Cdk1 activation [82]. Moreover, inhibiting PP1 with a specific inhibitor, Inhibitor-2, or with specific antibodies promotes Cdk1 activation in *Xenopus* cell-free systems or intact oocytes [83–85], whereas overexpressing PP1 delays M-phase entry [83]. Such a negative role of PP1 in Cdk1 activation could result from its ability to dephosphorylate the kinase Gwl at two autophosphorylation sites, S875 and S883, leading to the inactivation of this kinase that is further completed by the action of PP2A (see below) [86–88]. These apparently contradictory results regarding PP1 implication in the Cdk1 feedback loop could be explained by the fact that some of the PP1 isoforms would be insensitive to the various approaches aimed at suppressing its activity. Alternatively, these various isoforms could have different substrates, some of them targeting Cdc25 while others would target Gwl. They could as well be differentially regulated by their intracellular localization, some of them being active in a given compartment while others would be inhibited in another location of the cell. Given the current confusing vision concerning the roles of PP1 in Cdk1 activation, it is crucial to carry out a detailed analysis of this phosphatase, taking into consideration the composition of its different isoforms and their subcellular localization.

### Down-regulation of PP2A-B55δ

Beside regulating Cdc25 and Myt1, the Cdk1 auto-activation loop also requires that the phosphatases that oppose kinase activities, in particular that of Cdk1, are inhibited to ensure the effective phosphorylation of both Myt1 and Cdc25 and thus, their proper regulation during meiosis resumption. These phosphatases belong to the S/T phosphatase family that are sensitive to okadaic acid, a strong inhibitor whose injection in *Xenopus* oocytes activates Cdk1 independently of both protein synthesis and PKA activity [89]. Almost all these phosphatases are expressed in *Xenopus* oocytes: PP1, PP2A, PP4, PP5 and PP6 [90]. Clearly, the major Cdk1-counteracting phosphatase corresponds to PP2A [12]. Biochemical properties of PP2A are presented in Sidebar C.

**Table:** 

**Sidebar C. PP2A at a glance**
PP2A is a heterotrimer composed of a catalytic subunit (PP2A-C), a structural subunit (PP2A-A) and a regulatory subunit (PP2A-B). Eukaryotes have four B-subunit families known as B/B55 (PR55), B′/B56 (PR61), B″/B72 and B‴/Striatin, each of them comprising several very close isoforms whose number differs according the species [91]. Some of these different isoforms are specific to vertebrates, in particular the four α, β, γ and δ isoforms of B55 and the 5 isoforms α, β, γ, δ and ε of B56 [92]. The core part of PP2A is formed by the dimer PP2A-A/C and its association to a regulatory B-subunit drives both its substrate specificity and intracellular localization. Because of the multiple isoforms of PP2A subunits, hundreds of different holoenzymes are potentially generated that display very similar catalytic domains. Several mechanisms allow for the specific recognition of substrates by PP2A holoenzymes in vivo. Substrate binding involves specific consensus motifs: PP2A-B56 recognizes the SLiM “LxxIxE” while PP2A-B55 preferentially targets substrates with positively charged polybasic units on either side of the residue to be dephosphorylated [93–95]. Furthermore, phosphorylation of residues inside or adjacent to PP2A-B56 recognition motifs potentiates its binding to the substrate [94, 96].

In *Xenopus*, the isoform of PP2A that antagonizes Cdk1 activity corresponds to PP2A-B55δ, whose role and regulation are mostly conserved in all experimental models, both in mitosis and meiosis [15]. The activity of PP2A-B55 is tightly regulated during M-phase: it is active in interphase and is inactivated upon M-phase onset [12]. Its inactivation is achieved by Gwl kinase that phosphorylates two proteins of the Endosulfine family, Arpp19 and its paralog, α-endosulfine (ENSA) (Fig. 4) [97]. These proteins share highly conserved sequence similarity across most eukaryotes but likely display specific functions. Arpp19, but not ENSA, plays an essential role during mouse embryogenesis and in regulating mitotic and meiotic divisions [98]. ENSA specifically controls S-phase length in human cells by modulating the levels of the replication factor Treslin [99]. Gwl phosphorylates Arpp19 at S67 (*X. laevis* numbering) [34, 36], a residue located in the FDS_67_GDY motif, the most conserved region of the Endosulfine family [97]. Once phosphorylated at S67, ENSA/Arpp19 specifically binds to and inhibits PP2A-B55δ by titrating the phosphatase away from all other substrates (Fig. 4) [34, 36] [100]. In *Xenopus* oocytes, the activation of the Gwl/Arpp19 axis is critical for MPF autoamplification [37, 101]. Injection of an Arpp19 mutant, S67A, which cannot be phosphorylated by Gwl and acts as a dominant negative for Gwl activity, prevents Cdk1 activation in response to progesterone but also to any other trigger involved in the Cdk1 feedback loop [37]. Conversely, the injection of either Gwl (constitutively active or with a gain in activity as the K71M mutant) or a form of Arpp19 that cannot be dephosphorylated at S67, S67-thiophosphorylated Arpp19, is sufficient to trigger Cdk1 activation without progesterone [37, 101]. S67-thiophosphorylated Arpp19 constitutively interacts with and inhibits PP2A-B55δ, phenocopying the in vivo inhibition of S/T phosphatases by okadaic acid [37]. Hence, PP2A-B55δ inactivation by the Gwl-Arpp19 axis is both necessary and sufficient for Cdk1 activation in *Xenopus* oocytes (Fig. 4). Released from the activity of its counteracting enzyme, PP2A-B55δ, Cdk1 can phosphorylate Myt1, Cdc25 and its other M-phase substrates. Importantly, Gwl-Arpp19 activation and the subsequent PP2A-B55δ inhibition require Cdk1 activity, either achieved by Cdk1-Cyclin B in *Xenopus* oocytes [37] and Cdk1-Cyclin A in human cells [9]. Therefore, Cdk1 not only phosphorylates its own activation module, Cdc25-Myt1, but it further indirectly inactivates its antagonizing protein phosphatase, PP2A-B55δ, through Gwl-Arpp19 (Fig. 4). Once this system is active, Cdk1 is abruptly and irreversibly activated, switching the cell into M-phase.

### Launching the positive feedback loop: what has the finger on the trigger?

Cdk1 itself is able to regulate Myt1, Cdc25 and indirectly PP2A-B55δ through Gwl, which makes the loop turning. But Cdk1 is obviously inactive before the loop is launched, since the purpose of this loop is precisely to activate it. This egg-and-chicken model raises the question of what is the flip-flop switch that triggers the initiation of the Cdk1 circuit. The triggering mechanism must target few molecules of Cdc25 and Myt1, to activate the first ones and to inactivate the second ones. This implies (i) to recruit kinases able to phosphorylate Cdc25 and Myt1 and (ii) to inhibit PP2A-B55δ. Although the signalling pathway induced by progesterone and leading to Cdk1 activation remains unclear, it promotes the synthesis of new proteins from stockpiled mRNA, necessary for the meiosis resumption. Some of these newly synthesized proteins could activate Cdc25 or inhibit Myt1, causing the activation of the auto-amplification loop.

Such a candidate exists in Mos, a S/T kinase, the synthesis of which is upregulated in response to progesterone [102]. Mos is a direct activator of MAPK kinase (MEK), which in turn activates MAPK [103]. Injection of Mos or constitutively active forms of its downstream kinases induces meiotic division in the absence of progesterone [64–66, 102]. Moreover, it has been shown that Myt1 is inhibited by either Mos or a MAPK substrate, p90^Rsk^ [54, 56], providing a link between the Mos/MAPK cascade and Cdk1 activation. However, several studies convincingly showed that neither the activation of MAPK nor Mos synthesis is necessary for progesterone-induced MPF activation in *Xenopus* oocytes [104–106]. Moreover, the accumulation of Mos protein requires a stabilizing phosphorylation catalyzed by Cdk1 [107]. Consequently, the Mos/MAPK activation only takes place when Cdk1 activation is already initiated. It could undoubtedly contribute to the auto-activating Cdk1 circuit but cannot be its trigger. It should be noted that another kinase, the polo-like kinase Plx1, is able to phosphorylate and to inactivate Myt1, as well as controlling Cdc25 activation [38, 46, 50, 108]. However, its action towards Myt1 takes place during the embryonic mitotic cell cycles that follow fertilization but not during the meiotic cycle [46]. Moreover, Plx1 activation depends on Cdk1 [38], excluding a role of Plx1 as a trigger of the auto-amplification loop in *Xenopus* oocytes.

Another candidate is Cdk1 itself. This hypothesis relies on the formation of few active Cdk1 molecules, independently of the inactive pre-MPF stockpile, able to fire the loop by targeting Myt1, Cdc25 and PP2A-B55δ. Interestingly, a non-Cyclin protein, named Ringo/Speedy, accumulates transiently upon progesterone stimulation, binds and activates Cdk1 [109, 110]. Ringo-activated Cdk1 can phosphorylate and inhibit Myt1, supporting a role for Ringo in the MPF triggering mechanism [111]. Interestingly also, de novo synthesis of Cyclins is induced by progesterone [61, 112, 113]. G2-arrested oocytes contain a stockpile of Cyclins B2 and B5, two very similar proteins that are associated with Y15-T14-phosphorylated Cdk1 and form the inactive pre-MPF. In contrast, two other similar Cyclins, B1 and B4, are expressed at very low levels in the resting oocyte and accumulate in response to progesterone [113]. In contrast to Mos, the accumulation of Cyclin B1 promoted by progesterone is independent of MPF activity [114]. The other mitotic Cyclin type, Cyclin A, was shown by antisense experiments not to be required for oocyte maturation [115]. Moreover, Cyclin A is present at less than 1/100^th^ of the amount of Cyclin B and can be considered as effectively absent in *Xenopus* oocytes [112]. This excludes Cyclin A as playing a role in the triggering mechanism of the Cdk1 circuitry, in contrast to somatic cells where Cyclin A would be instrumental to control the mitotic entry threshold for Cdk1 activation [6–8]. Therefore, if binding of a Cyclin partner is critical for allosteric activation of Cdk1 in *Xenopus* oocytes, Cyclin B, and more precisely Cyclin B1, is the most attractive candidate.

**Fig. 5:**
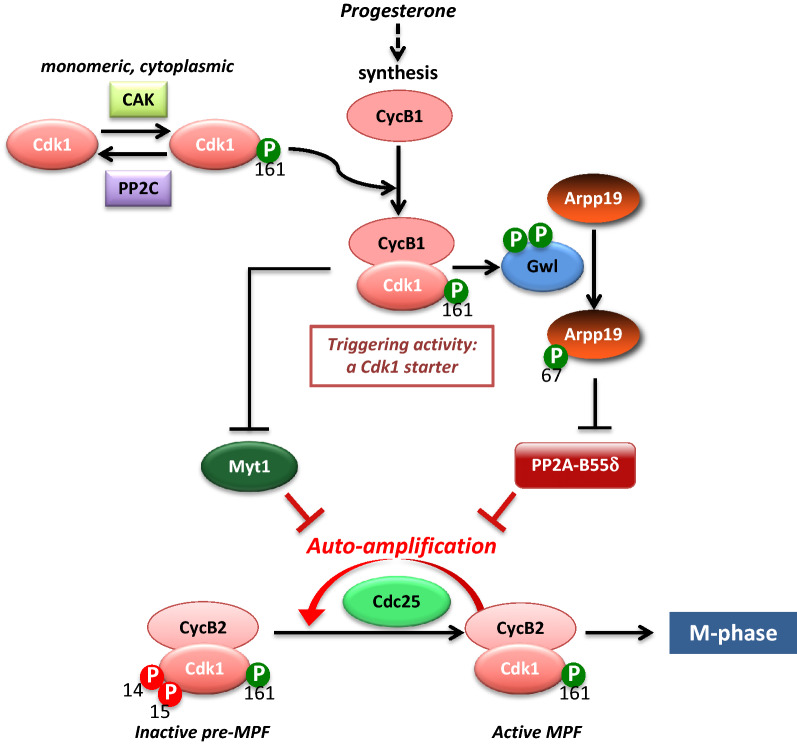
Generating a Cdk1 starter to launch the Cdk1 feedback loop. In response to progesterone, Cyclin B1 accumulates, binds monomeric Cdk1 and forms Cdk1-Cyclin B1 complexes that escape Myt1 inhibition and cause inactivating phosphorylation of Myt1. Hence, these few active complexes serve as a switch to initiate the MPF activation loop from the stockpiled Cdk1-Cyclin B2 complexes

Most of the Cdk1 protein is present as a monomer in the cytoplasm of the *Xenopus* oocyte, whereas a minor fraction is associated with B2 and B5 Cyclins, forming the inactive pre-MPF [113]. Hence, by binding and directly activating monomeric Cdk1, the new synthesized B1 Cyclin molecules could generate few active Cdk1 complexes that could act as a trigger, starting to reverse the balance of Cdc25 and Myt1 activities (Fig. 5).

Generating active Cdk1-Cyclin B1 requires Cdk1 to be phosphorylated at T161 and to escape Myt1 inhibitory phosphorylations. In vertebrates, T161 phosphorylation of Cdk1 is catalyzed by a CDK-activating kinase (CAK), composed of Cdk7, Cyclin H and the assembly factor MAT1 and strictly located in the nucleus [116, 117]. Cdk7-Cyclin H exhibits a stronger affinity for Cyclin-associated Cdks than for monomeric Cdks [118]. Surprisingly, *Xenopus* oocytes contain a significant amount of monomeric Cdk1 phosphorylated at T161 [119]. Moreover, injection of Cyclin B in enucleated oocytes leads to active Cdk1 complexes. These observations imply that Cyclin-free Cdk1 is a substrate of a cytoplasmic CAK distinct from Cdk7-Cyclin H.

In *Saccharomyces cerevisiae*, the only known CAK is a cytoplasmic monomeric enzyme [120–122]. In contrast to the Cdk7-Cyclin H complex, it preferentially phosphorylates monomeric Cdks rather than Cyclin-associated Cdks [121]. A monomeric CAK activity has been also detected in human cells [123, 124]. Such an enzyme could be responsible on T161 phosphorylation of monomeric Cdk1 in the *Xenopus* oocyte. The opposite phosphatase that specifically dephosphorylates Cdk1 at T161 in *Xenopus* oocytes has been identified as the Mg^2+^-dependent phosphatase, PP2C [119], as it is the case in budding yeast where the two major type 2C phosphatases dephosphorylate the Cdk1 yeast homolog, CDC28 [125]. Sidebar D summarizes the main biochemical features of PP2C. PP2C is unable to dephosphorylate Cyclin-associated Cdk1 and exhibits a strict affinity for monomeric Cdk1 [119]. Thus, a specific and still unknown regulation, implying an unidentified T161 kinase and its opposing phosphatase, PP2C, allows the presence of two monomeric Cdk1 subpopulations in the oocyte, one being phosphorylated on T161 while the other one is not (Fig. 5). Interestingly, monomeric Cdk1 molecules already phosphorylated at T161 represent a latent form of Cdk1 directly activable by Cyclin binding, without the requirement of phosphorylation by any CAK. This favors the formation of directly active new complexes between Cdk1 and newly synthesized Cyclin B1 acting as trigger of the Cdk1 auto-amplification loop. This hypothesis has been confirmed by the discovery that the critical event at the G2-to-M transition is a change in the balance between Cyclin B1 synthesis and Myt1 activity (Fig. 5) [49]. In G2-arrested oocytes, accumulation of B2 and B5 Cyclins is slow and newly formed Cdk1-Cyclin B complexes are immediately inactivated by Myt1, increasing the inactive pre-MPF population. In response to progesterone, stimulation of Cyclin B1 translation leads to newly formed Cdk1-Cyclin B complexes that escape Myt1 inhibition and cause rapid inactivating phosphorylation of Myt1, ahead of Cdc25 phosphorylation and activation, without any significant contribution of Mos/MAPK or Plx1 (Polo- like kinase 1 of *Xenopus*) [49]. This serves as a switch to initiate the MPF activation loop (Fig. 5).

**Table:** 

**Sidebar D. PP2C at a glance**
PP2C is the unique member of the family of Mg^2+^/Mn^2+^-dependent protein phosphatases [126]. It is found in both eukaryotes and some prokaryotes. PP2C is monomeric with two structural domains, a conserved N-terminal catalytic domain and a C-terminal region with substantial structural and sequence variance among different isoforms. The residues and the folding pattern of the active-site are highly conserved among eukaryotes, with two central five-stranded β-sheets sandwiched by two pairs of α-helices. The catalytic site is located at the edge of the two central β-sheets and contains 3 atoms of either Mn^2+^ or Mg^2+^ ions. In metazoa, gene duplication has led to functional diversification through many isoforms of PP2C (18 in mammalian cells) that have gained specificity for various signalling pathways and tissue expression patterns. These isoforms have been implicated in the regulation of stress signalling cascades, phosphatidylinositol 3-kinase/Akt signalling, pre-mRNA splicing, protein ubiquitination and degradation as well as cell metabolism, highlighting the role of PP2C in controlling cell differentiation, proliferation, growth and survival/death. However, little is known about the regulatory mechanisms of PP2C at the molecular level. Special emphasis should be placed on plants, in which PP2C represents the major group of protein phosphatases and is at the center stage of the major signalling pathways controlled by abscisic acid and regulating plant responses to stresses as well as plant growth and development [127].

How the new Cdk1-Cyclin B1 complexes escape Myt1 inhibitory phosphorylations is unknown. The “escape” could be explained by the titration of Myt1 kinase due to increasing quantities of new Cdk1-Cyclin B1 complexes and/or because the newly formed complexes are biochemically different (including Cyclin B1 and not Cyclin B2) or differently localized. The few Cdk1 activity generated by Cyclin B1 synthesis should be able to phosphorylate and activate Gwl, and consequently to decrease PP2A-B55δ activity through Arpp19 phosphorylation, meeting the second request of a triggering mechanism. It has been proposed that another partner of Cdk1, the non-Cyclin protein Ringo that is synthesized in response to progesterone as Cyclin B1 [109, 110], is also able to phosphorylate and to inhibit Myt1 by targeting different residues than those phosphorylated by Cdk1-Cyclin B [31, 111]. Moreover, Ringo-bound Cdk1 is more resistant than Cdk1-Cyclin B complexes to negative regulation via inhibitory phosphorylations catalyzed by Myt1 [128]. Hence, a complementary action of both new synthesized Cyclin B1 and Ringo could produce the small amount of Cdk1 activity that triggers the feedback loop.

### At the top of the signaling pathway: the critical dephosphorylation of Arpp19

**Fig. 6:**
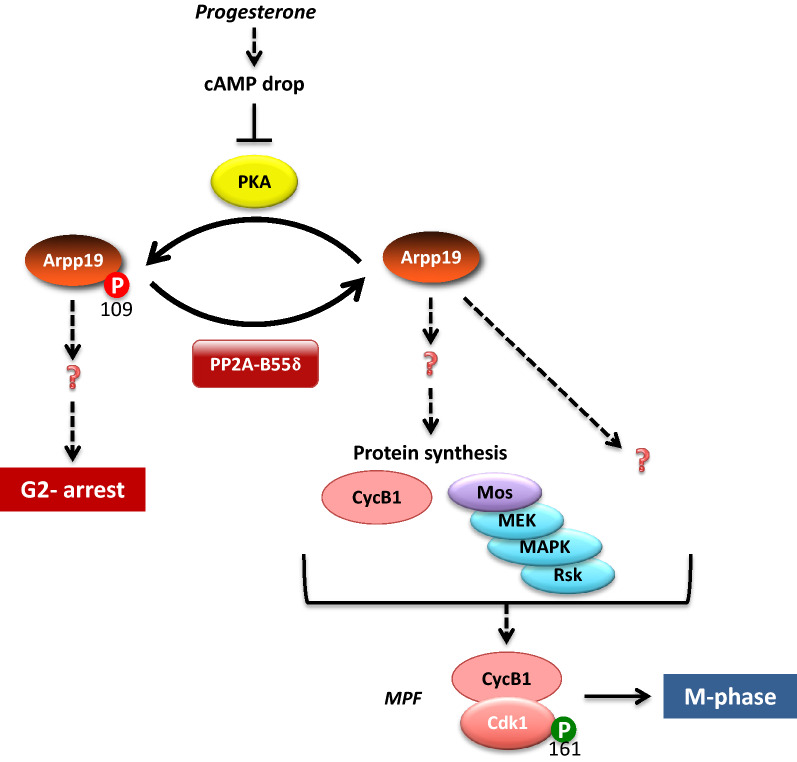
At the top of the signalling pathway, the critical Arpp19 dephosphorylation at S109 by PP2A-B55δ. The G2 lock is achieved by high cAMP, high PKA activity and Arpp19 phosphorylation at S109. Arpp19 phosphorylation turns over due to PKA and PP2A-B55δ, with PP2A-B55δ being swamped by PKA. Upon PKA inhibition promoted by progesterone, Arpp19 is dephosphorylated at S109 by PP2A-B55δ and launches the signalling pathway that ends with Cdk1 activation. The targets of Arpp19 are still unknown. It could control the synthesis of new proteins required to activate Cdk1, such as Cyclin B1 or Mos

While phosphatases are critical players of Cdk1 activation, which represents the endpoint of the signalling pathway induced by progesterone, the very beginning of the process is as well controlled by a specific dephosphorylation event that triggers the initiation of the signalling pathway. In all vertebrate oocytes, the lock in G2 is achieved by a high concentration of cAMP, which maintains a high level of PKA activity [26]. How the cAMP-PKA module locks the cell in G2, independently of DNA damage and replication checkpoints, is unknown. Progesterone induces within 10 to 30 min a drop in cAMP concentration, leading to PKA downregulation that launches a 3-to-5-hour-long signalling pathway whose endpoint is Cdk1 activation. Understanding how PKA controls the G2-to-M transition in oocytes, requires to identify its physiological substrates whose dephosphorylation, which occurs early in response to progesterone, initiates the molecular cascade ending with Cdk1 activation. Few PKA targets have been proposed to block oocyte meiosis resumption, including two direct regulators of Cdk1, Wee1 and Cdc25 [72, 129]. Although Wee1 is a key MPF inhibitory kinase in mouse oocytes, functioning downstream of PKA [129], this protein is not expressed in *Xenopus* oocytes [19]. As previously discussed, another candidate is Cdc25, which is phosphorylated at S287 by PKA in *Xenopus* oocyte, a phosphorylation that would impair its activation and could contribute to the G2 arrest [72]. However, S287 dephosphorylation, presumably catalyzed by PP1 [73], occurs at the time of Cdk1 activation, few hours after PKA downregulation. Hence, this late dephosphorylation cannot account for the launching of the signalling pathway that is initiated within 30 min in response to progesterone. In an attempt to search for the PKA substrate responsible for the oocyte G2 arrest, a 20 kDa phosphoprotein was partially purified from *Xenopus* oocytes by virtue of its acid solubility and thermostability [130] but the molecular identity of this protein was not further analyzed. Almost 30 years later, a protein with similar biochemical characteristics was discovered to be a crucial PKA substrate in *Xenopus* oocytes. Very surprisingly, this early PKA substrate is Arpp19, which plays an important role in the Cdk1 auto-activating circuit by inhibiting PP2A-B55δ thanks to its phosphorylation at S67 by Gwl (Fig. 4) [131]. In *Xenopus* G2-arrested oocytes, PKA phosphorylates Arpp19 at another residue, S109, and this phosphorylation is important for the G2 lock (Fig. 6) [28]. Upon progesterone stimulation, the decrease in PKA activity leads to Arpp19 partial dephosphorylation at S109 within half an hour. The role of S109 phosphorylation was enlightened by injecting a phosphomimic mutant of Arpp19, Arpp19-S109D, which fully inhibits Cdk1 activation and M-phase entry [28]. However, this S109 phosphomimic mutant is not able to inhibit the Cdk1 auto-amplification loop when this process is directly launched in the oocyte by injecting any component of the loop [28]. Hence, Arpp19 dephosphorylation at S109 is critical to unlock the G2 arrest by initiating a few-hour-long molecular cascade that ultimately ends with Cdk1 activation. However, the phosphorylation of this S109 residue does not control the Cdk1 auto-amplification loop. The shift is taken by the Gwl-dependent phosphorylation of Arpp19 at S67 that is essential for the Cdk1 feedback loop. Hence, S109-phosphorylated Arpp19 locks the oocyte in G2 whereas the S67-phosphorylated form is necessary for M-phase entry [131].

The oocyte G2 release therefore relies on a specific phosphatase that dephosphorylates Arpp19 at S109. Using a biochemical chromatography procedure combined with mass spectrometry sequencing, our team just discovered that this phosphatase corresponds to PP2A-B55δ (Fig. 6) [90]. The specific inhibition of PP2A-B55δ in intact oocytes fully abolishes the S109 dephosphorylation of Arpp19 upon progesterone addition [90]. Therefore, just as Arpp19 that plays a dual role in the oocyte at both extremities of the signalling cascade, the same phosphatase, PP2A-B55δ, also controls the starting point as well as the Cdk1 final point, being required to dephosphorylate Arpp19 at S109 at the beginning, and inhibited by S67-phosphorylated Arpp19 at the end.

Interestingly, PP2A-B55δ is active in G2-arrested oocytes, implying that Arpp19 phosphorylation at S109 turns over thanks to an equilibrium between PKA and PP2A-B55δ activities, with the action of PP2A-B55δ being swamped by PKA. Both antagonistic enzymes do not cross-regulate each other in this system. In contrast to PKA, whose activity decreases in response to progesterone, PP2A-B55δ stays active. As a consequence, upon PKA inhibition promoted by progesterone, Arpp19 is dephosphorylated at S109 by PP2A-B55δ and launches the signalling pathway that ultimately ends with Cdk1 activation (Fig. 6). These results indicate that the G2 arrest of the oocyte is a highly dynamic process during which PKA and PP2A-B55δ are engaged in a futile cycle of phosphorylation/dephosphorylation regarding their common substrate, Arpp19. This contrasts with productive cycles characterized by two enzymes that operate alternatively and communicate with each other, avoiding ATP waste. Such a futile cycle allows both opposed active enzymes to carry important functions independently of each other. As such, PKA certainly targets substrates other than Arpp19, important to keep oocytes arrested in prophase, such as Cdc25 or Wee1 in mouse oocytes [72, 129]. PKA activity also negatively and indirectly regulates Gwl activity, as reported in *Xenopus* oocytes and yeast [37, 132]. Meantime, the sustained activity of PP2A-B55δ also ensures the stability of the G2 arrest by impeding Cdk1 activation and its substrates phosphorylation. Besides preventing phosphorylations of Cdc25 and Myt1, PP2A-B55δ avoids also Arpp19 phosphorylation at S67 by Gwl [100], preserving its own activity and preventing premature Cdk1 activation.

The molecular mechanism controlled by Arpp19 phosphorylation at S109 has not been yet elucidated. Arpp19 phosphorylation at S109 could block its own phosphorylation at S67 and/or impede PP2A-B55δ inhibition. Such mechanism has already been described in human brain. In striatum neurons, Arpp16, the alternatively spliced variant of Arpp19, is phosphorylated by the human Gwl homolog, the Mast3 kinase, at S46 (equivalent to S67 in *Xenopus*) and strongly inhibits PP2A-B55δ [133]. In response to dopamine, PKA is activated and phosphorylates Arpp16 at S88 (equivalent to S109 in *Xenopus*) [134]. Interestingly, this phosphorylation renders Arpp16 non-phosphorylable at S46 by Mast3 and makes PP2A-B55δ non-inhibitable [135]. Such mechanism is unlikely functional in *Xenopus* as Gwl in vitro phosphorylates Arpp19 at S67 independently of S109 phosphorylation [136]. Moreover, during oocyte meiotic resumption, after being partially dephosphorylated at S109 by PP2A-B55δ, Arpp19 is then rephosphorylated at S109 at the time of Cdk1 activation, concomitantly with its phosphorylation at S67 by Gwl [28], excluding any biochemical and functional antagonism between S109 and S67 phosphorylations. Another attractive hypothesis is that S109-phosphorylated Arpp19 would block the synthesis of new proteins required to activate Cdk1, such as Cyclin B1 or Mos. Indeed, it has been reported that Arpp19 is involved in the regulation of translation as well as mRNA stabilization in human cells and yeast [137, 138]. Alternatively, by mirroring the function of S67-phosphorylated Arpp19, S109-phosphorylated Arpp19 could regulate an important phosphatase distinct from PP2A-B55δ. As such, PP1 is an interesting candidate. Although the implication of PP1 in the Cdk1 auto-amplification loop is far to be understood (see above), this phosphatase plays a positive role at the early steps of meiosis resumption in *Xenopus* oocytes, upstream Cdk1 activation [139]. Arpp19 could behave as a PP1 inhibitor when phosphorylated by PKA, similarly to DARPP-32, another PKA substrate, which is converted into an inhibitor of PP1 when phosphorylated by PKA [140]. Although neither Arpp-19 nor ENSA inhibited PP1 in in vitro assays, regardless of their phosphorylation state [36], further investigations using biological in vivo systems would be worthwhile.

## Conclusions

The discoveries on the G2-to-M release from the last decade have changed our vision of this important step of the cell cycle by highlighting the crucial importance of coordinating phosphatases with opposing kinases. The historical *Xenopus* oocyte model has further proven its powerfulness as a paradigm for the study of the G2-to-M transition. Resumption of meiosis was considered to be under control of two main kinases: first, PKA whose activity determines the G2 block when high and the G2 release when low; second, Cdk1 whose activity promotes M-phase. The first conceptual shift arose from the observation that not only is Cdk1 activated but that its antagonizing protein phosphatase, PP2A-B55δ, needs also to be inhibited for entry and progression through M-phase. Hence, M-phase entry is not viewed anymore as under the control of a single master kinase, but of a couple of two antagonistic enzymes whose regulation is of paramount importance. A second surprise was to find that the phosphatase that opposes Cdk1 and prevents M-phase onset, PP2A-B55δ, also opposes PKA and prevents the G2 arrest: a kind of opposite roles played by this phosphatase a few hours apart during meiosis resumption, establishing PP2A-B55δ as the main orchestrator of the oocyte G2-to-M transition.

However, the involvement of PP2A at both ends of the signalling pathway, which begins with Arpp19 dephosphorylation at S109 and ends with its phosphorylation at S67, is part of very different dynamic cycles. PP2A-B55δ faces Cdk1 and Gwl, through a productive cycle of phosphorylation/dephosphorylation. The phosphatase and its opposing kinases work alternatively. More, they reciprocally regulate each other, with inhibition of the phosphatase when kinases are active and activation of the phosphatase when kinase activities are decreased. Such a black or white situation avoids a waste of ATP. It also ensures a full switch-like interconversion of the phosphorylation state of the substrates, from an unphosphorylated state to a heavily phosphorylated state, resulting in the rapid and irreversible cell decision to enter M-phase. In strong contrast to the onset of M-phase, the hallmark of the oocyte G2 arrest relies on the simultaneous activities of both mutually antagonistic enzymes, PP2A-B55δ and PKA. Importantly, both enzymes do not regulate each other. Such a futile cycle renders impossible a full switch of their common substrate phosphorylation. Indeed, this fits with the observation that only half of Arpp19 is dephosphorylated at S109 in response to progesterone [28]. Further investigation will be necessary to understand how half of dephosphorylated Arpp19 can carry out its function despite the presence of the S109-phosphorylated Arpp19. The other consequence of maintaining a futile cycle is that both opposed active enzymes can carry important functions independently of each other by targeting non-common substrates, thus contributing to the G2 block by alternative mechanisms.

Remarkably, both Arpp19 phosphorylation sites important for the control of meiosis resumption, S109 and S67, are dephosphorylated by a unique phosphatase, PP2A-B55δ. The function of Arpp19 phosphorylation at S67 in converting this protein into a PP2A-B55δ inhibitor is well documented, whereas the action of S109 phosphorylation of Arpp19 in ensuring the G2 arrest remains unknown and deserves investigation. As is also worth exploring the other phosphatases acting during meiotic division, especially the various forms of PP1 and PP2A, as well as their regulation. We obviously must continue to refine our vision of these crucial and complex enzymes.

## Data Availability

Not applicable

## Abbreviations

Arpp19: cAMP regulated phosphoprotein of 19 kDa
ATM: Ataxia-Telengiectasia
cAMP: cyclic adenosine monophosphate
CAK: Cdk activating kinase
Cdc25: Cell division cycle 25
Cdk1: Cyclin-dependent kinase 1
Chk1: Checkpoint kinase 1
ENSA: α-Endosulfine
GVBD: Germinal Vesicle Breakdown
Gwl: Greatwall kinase
MAPK: mitogen-activated protein kinase
MEK: MAPK/ERK kinase
MPF: M-phase Promoting Factor
Myt1: Membrane-associated tyrosine and threonine 1
p90^Rsk^: p90 ribosomal S6 kinase
pT: Phosphorylated Threonine
pY: phosphorylated tyrosine
Plx1: Polo-like kinase of *Xenopus*
PP1: Protein phosphatase 1
PP1c: PP1 catalytic subunit
PP2A: Protein phosphatase 2A
PP2A-A: PP2A structural subunit
PP2A-B: PP2A regulatory subunit
PP2A-C: PP2A catalytic subunit
PP2C: Protein phosphatase 2C
PIPs: PP1-interacting proteins
PKA: cAMP-dependent protein kinase
